# A Mechanistic Map of the Phenotypic Complexity in Recessive Dystrophic Epidermolysis Bullosa Provides Basis for Therapeutic Target Discovery and Drug Repurposing

**DOI:** 10.3390/ijms27198436

**Published:** 2026-09-22

**Authors:** Sara Herráiz-Gil, Miriam Payá-Milans, María del Carmen de Arriba, Lucía Martínez-Santamaría, Carlos Loucera, Nuria Illera, Marcela del Río, Joaquín Dopazo, Carlos León, María José Escámez

**Affiliations:** 1 Departamento de Bioingeniería, Universidad Carlos III de Madrid, ROR Code 03ths8210, Avenida de la Universidad, 30 (Edificio Sabatini), 28911 Leganés, Spain; sherraiz@ing.uc3m.es (S.H.-G.); lmsantam@ing.uc3m.es (L.M.-S.); mescamez@ing.uc3m.es (M.J.E.); 2 Centro de Investigación Biomédica en Red de Enfermedades Raras (CIBERER-ISCIII), 28029 Madrid, Spain; 3 Instituto de Investigación Sanitaria Fundación Jiménez Díaz (IIS-FJD, UAM), 28040 Madrid, Spain; 4 Centro de Investigaciones Energéticas, Medioambientales y Tecnológicas, 28040 Madrid, Spain; 5 Platform for Computational Medicine, Andalusian Public Foundation Progress and Health-FPS, 41092 Seville, Spain; 6 Computational Systems Medicine, Institute of Biomedicine of Seville (IBIS), Hospital Virgen del Rocío, 41013 Seville, Spain

**Keywords:** recessive dystrophic epidermolysis bullosa (RDEB), machine learning, mechanistic models, drug repurposing, transcriptomics data, skin biopsies

## Abstract

Recessive dystrophic epidermolysis bullosa (RDEB) is a genodermatosis caused by loss-of-function mutations in the COL7A1 gene, producing a complex and heterogeneous phenotype. Clinical manifestations include skin fragility, local and systemic inflammation, impaired wound healing, dysbiosis, pain, and pruritus, increasing the risk of skin cancer. This work aimed to develop an actionable model of RDEB’s molecular mechanisms to identify and prioritize therapeutic targets for drug repurposing. Transcriptomic data of skin biopsies from pediatric RDEB patients and unaffected individuals were used to build the mechanistic map of the disease. This model, combined with machine learning, made it possible to infer actionable targets from the map circuits and identify candidate drugs capable of reversing the transcriptomic alterations. The resulting map is defined by 13 altered signaling circuits and 10 effector proteins which were linked to disease hallmarks including inflammation, fibrosis, and tumor progression. Analysis of the map identified 66 actionable targets with 74 drug candidates. Among them, overrepresentation analysis showed enrichment of phosphodiesterases and antineoplastic and immunomodulating agents. Finally, 16 drugs were prioritized for their potential to revert circuit alterations without potentiating unintended effects, with bendroflumethiazide and ibutilide showing the highest impact. This mechanistic model delivers the first functional disease map of RDEB and reveals promising therapeutic candidates targeted by approved drugs.

## 1. Introduction

Epidermolysis bullosa (EB) is a heterogeneous group of low-prevalence genodermatoses characterized by spontaneous or minimal trauma-induced blistering [1,2]. Among them, recessive dystrophic EB (RDEB), caused by mutations in the COL7A1 gene, is one of the most severe subtypes [3]. This gene encodes type VII collagen (C7), the main component of anchoring fibrils which are essential for dermal–epidermal attachment. In RDEB, skin blistering is often linked to non-healing wounds and fibrosis [4], contributing to local and systemic inflammation [5], pseudosyndactyly, and a high risk of developing squamous cell carcinoma (SCC), the main cause of early mortality [6,7]. This loss of skin integrity and chronic tissue damage is accompanied by dysbiosis [8] and bacterial colonization [9], determinants of innate and adaptive immunity, which are also altered in RDEB [10,11]. Mucosal involvement complicates feeding and, together with systemic inflammation, results in malnutrition and anemia [12,13]. Additionally, peripheral neuropathology, also associated with inflammation, may contribute to pruritus and chronic pain [14,15], important factors impairing quality of life of people with RDEB [16].

Standard care for RDEB, ideally multidisciplinary and combined with psychosocial support, focuses on prevention and treatment of wounds and other clinical complications [13]. Several drugs are used to treat the most common symptoms [17,18,19]. However, their efficacy is limited and chronic use may lead to serious side effects. As these therapies are primarily symptomatic and marginally effective, a wide variety of advanced therapeutic strategies for RDEB, including cell and gene therapy, are under investigation [20]. Some of these therapies have been explored in clinical trials [2], but most are not yet incorporated into standard clinic routine. The approval of the first gene therapy approach, Vyjuvek (FDA, 2023; EMA, 2025), marked a milestone in RDEB treatment, followed by Zevaskyn (FDA, 2025). Vyjuvek provides transient in vivo correction of the COL7A1 gene through the use of non-integrative vectors [21], while Zevaskyn uses skin grafts with integrative vectors to achieve a permanent effect [22]. A third approved treatment is Filsuvez (EMA/FDA, 2023), a birch bark triterpene-based topical gel that promotes wound healing [23]. Despite these advances, unmet medical needs are reported by 40% of EB patients [24], underscoring the interest in developing or repurposing drugs to support their treatment [20,25].

Transcriptomic data exploration for drug repurposing in EB is gaining interest [26,27,28,29]. However, a better understanding of the subjacent molecular mechanisms remains essential to develop clinically relevant therapies. Mechanistic models, defined as mathematical representations of biological systems, provide a causal link between gene expression and functional cell behavior [30]. These models accurately infer causality and can predict the effects of gene perturbations such as drug impacts on pathway activities and downstream cellular functional consequences [31,32]. This approach has successfully predicted drug candidates for repurposing in rare diseases such as Fanconi anemia and retinitis pigmentosa, as well as in the COVID-19 pandemic [33,34,35,36]. Applying this mechanistic approach to RDEB is novel and may yield clinically relevant therapeutic candidates.

In this study, we developed an actionable functional model of RDEB, generating the first mechanistic map of the disease, based on transcriptomic data from pediatric patients. Using explainable machine learning models trained on publicly available gene expression data, we assessed the impact of existing drug targets on this RDEB signaling pathway map. This approach identified and prioritized therapeutic target genes suitable for drug repurposing in RDEB.

## 2. Results

### 2.1. Genotype and Clinical Features in the RDEB Pediatric Cohort

All patients were biallelic carriers of premature termination codon (PTC) variants with null or minimal C7 cutaneous expression and exhibited a severe phenotype (EBDASI > 107), despite variability in affected skin area, inflammatory markers, pain, and pruritus (Table S1).

### 2.2. Construction of the RDEB Mechanistic Map

RNA-Seq data from eight pediatric RDEB patients and six unaffected controls were analyzed to construct the RDEB skin mechanistic map. A total of 255 differentially expressed genes were detected (GSE119501, FDR < 0.05), and 68 significantly altered proteins were mapped to KEGG signaling pathways with 27 overexpressed and 41 underexpressed in the disease (Figure S1A–K). Functional enrichment analysis provided a list of 11 significant GO Biological Processes and 1 Uniprot Keyword (Table S2). Functions related to antiviral defense, cell adhesion, immune cell chemotaxis, GTPase activation and protein phosphorylation, and BMP/SMAD signaling were altered, along with processes that modulate cellular organization and the formation of protein complexes. These changes reflect a disruption of signaling networks relevant to inflammation and tissue remodeling in RDEB.

Mechanistic analysis predicted significant differential activity of 10 effector proteins, participating in 13 differentially activated circuits (DACs) and associated with 11 KEGG signaling pathways (FDR < 0.05, Table 1). Among the DACs, the map included nine stimulated circuits linked to eight final effectors (CCL21, SMAD7, GRK2, EZR, KCNJ1, IKK1, RNASEL, and CASP3), as well as four repressed circuits with EIF4EBP and ACTB as the two final effectors. Specific DACs are shown in Figure S1A–K.

The RDEB map identified effector proteins with functional contributions to well-established disease hallmarks (Figure 1). Most effectors contributed to inflammation, fibrosis, and tumor development. Additional associations included pruritus (GRK2), malnutrition (EIF4EBP), pain (IKK1, RNASEL), chronic wounds (CCL21, CASP3), immune response (EZR, KCNJ1, CCL21), dysbiosis (ACTB, RNASEL, IKK1), and skin integrity (EZR, KCNJ1, ACTB).

The predictive value of these connections was corroborated using patient-specific RNA-seq and clinical data. Pairwise correlations between hallmark features and effector gene expression (log2CPM) partially validated the map predictions (Figure 2). Circulating CRP negatively correlated with IKK1 (*p* = 0.011) and SMAD7 (*p* = 0.022), and positively with CASP3 (*p* = 0.015), confirming the predicted contribution of these effectors to inflammation. Disease severity (EBDASI) was positively associated with RNASEL (*p* = 0.045) and negatively with IKK1 (*p* = 0.004), in agreement with the map. Itch severity correlated positively with EIF4EBP (*p* = 0.01), supporting its predicted link to pruritus. Notably, there was a significant negative correlation between TGF-β1 and KCNJ1 mRNA levels (*p*-value = 0.041). However, no other effectors showed significant correlations with TGF-β and no significant differences were found at the TGF-β mRNA level between the two cohorts.

### 2.3. Prediction of Therapeutic Targets and Proposal of Repurposed Drugs for RDEB

Using the drexml machine learning strategy [37], 66 known drug targets (KDTs) were predicted as significantly impacting the 13 altered circuits in the RDEB mechanistic map (Figure 3). Each circuit included at least one KDT with high confidence (CI > 95%), and strong performance (R^2^ > 0.6) and stability (s > 0.4) (Figure 3A). The absolute SHAP values, reflecting the strength of the circuit–KDT interactions, highlighted the most influential targets (Figure 3B).

Potential RDEB drug candidates were prioritized using two strategies: (1) selecting the KDTs with highest SHAP values and performing MBRole enrichment, and (2) refining this selection with a recalculated score accounting for effects across other map circuits. The first approach linked the top 30 KDTs to 74 marketed drugs retrieved from DrugBank (Figure 4A). Information on these drugs is provided in Table S3 with their DrugBank and PubChem IDs. Relevant details, including the SHAP values associated with each circuit in the RDEB map, the ATC classification, the drug’s actual use, and the reported use in EB (if any), are highlighted in Table S3 for each of them. Notably, 19 of these 74 drugs are already in preclinical research or clinical use for treating EB (including 4 protein kinase inhibitors and 3 antineoplastic agents), supporting the relevance of the predictions. However, some of these drugs have exhibited limited specificity or only marginal efficacy.

A functional enrichment analysis on the 74 drugs, performed with MBrole 3.0, provided a list of enriched KEGG drug classification and ATC codes (Table S4). Interestingly, the most enriched KEGG drug terms were related to the phosphodiesterase family (including PDE5 and PDE3) and receptor tyrosine kinases (Figure 4B). Other relevant groups included drugs targeting platelet-derived growth factor (PDGFR) and vascular endothelial growth factor receptor (VEGFR) drug families. Likewise, ATC code functional analyses (Figure 4C) revealed an overrepresentation of drugs with therapeutic actions such as protein kinase inhibitors, antineoplastic and immunomodulating agents, and PDE inhibitors.

This initial drug selection strategy, while effective, may identify drugs with unintended effects on the circuits of the disease map. To refine this selection, the second strategy was based on both the recalculated drug score and the percentage of circuits affected. The Elbow method (Figure 5A) suggested three clusters, with Group 2 including 16 drugs with mean recalculated scores closer to −1 (Figure 5B), highlighting them as optimal for further analysis. Figure 5C highlights the specific RDEB map circuits targeted by Group 2 drugs and Table S5 provides detailed information, including mechanism of action, SHAP value, percentage of affected circuits, approved indications, and reported use in EB. Among these 16 drugs, sucralfate, hydrocortisone, and phenytoin are already used for EB (including RDEB) reinforcing the reliability of the results. Finally, three drugs in Group 2, namely ibutilide, sucralfate, and bendroflumethiazide, show a higher impact over the map circuits in terms of percentage of circuits affected (Figure 5C). While only sucralfate and bendroflumethiazide are among the ones targeted by the top 30 KDTs (Figure 5D), the potential impact of these drugs on the hallmarks of RDEB, estimated through the normalized SHAP values of their corresponding protein targets, is shown in Figure 5E. Sucralfate, ibutilide, and bendroflumethiazide are associated with the reversal of altered circuits in RDEB, mainly modulating hallmarks such as inflammatory response and tumor development, but also others such as skin integrity, dysbiosis, wound healing, and immune response (Figure 5E).

## 3. Discussion

Drug repositioning represents an attractive option for rare diseases such as RDEB, where standard management remains largely symptomatic and traditional drug development is constrained by limited market incentives despite orphan drug designation. In this scenario, mechanistic models aiming to understand disease pathology and progression are key to refining therapeutic strategies. In this work, RNA-Seq data from skin biopsies of a pediatric RDEB cohort were used to build a mechanistic map of the disease. This map provides a precise view of the RDEB signaling landscape by predicting the differential activity of circuits (DACs) and establishing their functional links with known pathological hallmarks. In addition, DACs constituting the map were used as inputs to identify actionable targets and drug candidates with the potential to reverse the circuit signature. To date, three transcriptomic-based drug repurposing studies in EB [26,27,28] have identified and partially validated drugs using databases from cell lines which include both approved drugs and compounds in early development. However, none of them applied this novel mechanistic approach, which provides a framework that offers a set of clinically approved drug candidates with high translational potential.

### 3.1. RDEB Mechanistic Map

The RDEB map predictions covered a broad range of disease hallmarks. Most of the effectors from the 13 DACs were linked to inflammation, fibrosis, and tumor development, while some of them were also related to pruritus (GRK2), malnutrition (EIF4EBP), pain (IKK1, RNASEL), chronic wounds (CCL21, CASP3), immune response (EZR, KCNJ1, CCL21), dysbiosis (ACTB, RNASEL, IKK1), and impaired skin integrity (EZR, KCNJ1, ACTB). Correlations between patient-specific levels of RNA, biochemical parameters, and clinical data further support the predictions proposed by the map. These observations suggest that IKK1 and SMAD7 may act as negative regulators of inflammation, whereas CASP3 and RNASEL may contribute to inflammatory responses and disease severity. EIF4EBP may be involved in pruritus, and the inverse correlation between TGF-β1 and KCNJ1 suggests a potential feedback mechanism modulating TGF-β-driven pathways.

TGF-β, a well-known therapeutic target to attenuate inflammation, fibrosis, and potentially prevent SCC in RDEB [18], did not appear directly in the mechanistic map in this pediatric cohort. Nevertheless, total circulating TGF-β levels were significantly, but modestly, elevated in RDEB patients compared to controls (median IQR: 4.5 × 10^6^ vs. 3.7 × 10^4^, *p* = 0.033), while TGF-β transcripts in skin did not differ significantly. This apparent discrepancy likely reflects complex post-transcriptional and protein-level regulation of TGF-β, including proteolytic activation, latent complex release, and compartment-specific activity [38,39]. In addition, baseline TGF-β levels are naturally higher in children than in adults [40], which may mask differences between pediatric RDEB patients and controls. Furthermore, similar modestly elevated serum levels of TGF-β have been observed in another EB cohort [41], suggesting that the pediatric context may influence interpretation of this biomarker. Indeed, several circuits identified in the map are functionally connected to TGF-β activity. These include SMAD7 and IKK1 transcriptional regulation, NF-κβ signaling pathway, and CASP3-mediated apoptosis.

In this cohort, transcript levels of the effector IKK1 in skin showed significant inverse correlations with both inflammation (CRP) and disease severity (EBDASI). TGF-β signaling contributes to NF-κB activation, leading to upregulation of the effector CCL21, which mediates immune cell recruitment and is implicated in cutaneous inflammation and cancer metastasis [42,43,44,45]. Additionally, IKK1, a modulator of the NF-κB pathway, regulates CASP3-mediated apoptosis [46]. CASP3 activation in the RDEB map, which was positively correlated with CRP in this cohort, has been shown to increase lysophosphocholine and arachidonic acid (AA) release [47]. AA is a precursor of prostaglandin E2 (PGE-2), which plays a key role in stem cell proliferation, tissue regeneration, and wound healing, but can also contribute to inflammatory disorders. The combination of chemotherapy or immunotherapy with AA metabolic pathway inhibitors represents an effective strategy for managing inflammation in clinical settings [48]. Elevated circulating AA in plasma and erythrocytes has been previously associated with EB pathology [49].

Furthermore, IKK proteins are related to SMAD7-inhibited signaling in inflammatory processes [50]. Both SMAD7 and GRK2, TGF-β pathway antagonists [51,52], are activated in the RDEB skin map. Overexpression of these antagonists may represent a compensatory anti-fibrotic mechanism, which is consistent with SMAD7’s negative correlation with CRP in this pediatric cohort. A dual effect of SMAD7 has been associated with either attenuation or progression of diseases, depending on tissue type, cellular environment, inflammatory status, and disease stage [50,51]. Similarly, GRK2 activation is linked to several RDEB hallmarks, including fibrosis likely via NF-κβ signaling, tumor progression, and pruritus [53,54,55]. Also, GRK2 mediates phosphorylation of ezrin [56], a marker for fibrosis and cancer induced by TGF-β [57], that is likewise activated in the RDEB map. Recent studies also highlight the importance of TGF-β in conjunction with ion transporters such as KCNJ1, which may influence MHCI degradation and immune evasion [58]. Notably, KCNJ1 and TGF-β1 transcript levels were inversely correlated, suggesting a feedback regulatory interaction within the skin microenvironment in RDEB. The role of KCNJ1, CASP3, and ezrin in EB remains unexplored, underscoring the relevance of these findings.

In addition to TGF-β-related pathways, the Herpes simplex infection and Influenza A pathways are both activated in the RDEB map, converging on RNASEL, whose transcript levels in the skin positively correlate with disease severity in this cohort. RNASEL has been linked to tumorigenesis and innate immune responses [59], balancing tissue regeneration with antiviral capabilities [60], and may represent a target in RDEB independently of TGF-β. Supporting this, a recent screen of FDA-approved drugs for RDEB showed promising results of antiviral agents, improving fibrotic traits as well as malnutrition and pruritus in a mouse model [29].

In summary, the mechanistic map captures known drivers of RDEB and reveals novel molecular effectors (KCNJ1, CASP3, and ezrin), offering insights into its pathophysiology and pointing to new potential therapeutic targets, as also explored in this work. The observed correlations with clinical parameters support the map’s predictions and are consistent with previous research, but their generalizability should be confirmed in independent cohorts.

### 3.2. Prediction of Therapeutic Targets and Drug Candidates for Repurposing in RDEB

The drexml-based analysis of the RDEB mechanistic map identified 66 KDTs linked to 74 marketed drugs, 19 of which are already used in RDEB or other EB types, supporting the relevance of the predictions. While some of these drugs currently used in RDEB are non-specific or have shown limited efficacy, their identification may reflect the complexity of the disease and the multiple pathways involved, rather than a weakness of the prediction approach. The drug score provides a quantitative measure to prioritize candidates with stronger predicted effects on the altered circuits.

Enrichment analysis highlighted antineoplastic and immunomodulatory classes, as well as drugs targeting the VEGFR family, which interrupt signaling pathways involved in tumor progression, including metastasis [61]. Other overrepresented drug classes include phosphodiesterase inhibitors (PDE3 and PDE5) and protein kinase inhibitors. Among these, EGFR antagonists and tyrosine kinase inhibitors may provide palliation in advanced EB-associated SCCs [13]. Also, apremilast, a PDE4 inhibitor, has been used to treat EB Simplex [25].

The applied machine learning strategy, combined with a method to minimize unintended effects on the map circuits, led to a refined list of 16 candidate drugs including sucralfate, hydrocortisone, and phenytoin, which have already been used in EB [62,63,64]. Among the other 13 novel candidate drugs, 3 Bruton’s tyrosine kinase (BTK) inhibitors (acalabrutinib, ibrutinib, and zanubrutinib) were identified. Some BTK inhibitors are associated with improved wound healing and reduced proinflammatory states [65]. Specifically, ibrutinib reduces blistering in paraneoplastic bullous pemphigoid [66]. Histone deacetylase (HDAC) inhibitors such as romidepsin and vorinostat were also identified within the candidate drugs. HDAC inhibitors are employed to treat fibrosis and pruritus in patients with cutaneous T-cell lymphoma [67]. Notably, RDEB skin exhibits histone hypoacetylation, and systemic treatment with the HDAC inhibitor sodium valproate ameliorates disease in RDEB mice [68], supporting the therapeutic potential of targeting epigenetic modifications. Finally, tipiracil is a drug directed against thymidine phosphorylase, an enzyme overexpressed in SCC and associated with increased tumor aggressiveness and invasiveness [69]. The impact of this enzyme on psoriasis and rheumatoid arthritis has been described [70,71] and therefore tipiracil could be a good candidate to modulate inflammation in RDEB.

Finally, bendroflumethiazide and ibutilide showed the highest impact on the map circuits. Bendroflumethiazide, a diuretic commonly used for familial hyperkalemia, hypertension, edema, and urinary tract disorders, is considered safer than other thiazides with regard to skin cancer risk [72]. Its predicted mechanism involves activation of KCNMA1, which protects against TNFα-induced inflammation and interacts with integrins and cytoskeletal proteins to favor wound repair [73]. Ibutilide, a class III antiarrhythmic for atrial fibrillation and flutter, modulates cellular electrical activity via sodium, calcium, and potassium channels, which are also involved in pruritus, pain, fibrosis, and tumor progression [74,75,76]. Its predicted target, CACNA1C, functions as an immune checkpoint inhibitor in cutaneous melanoma and has been reported as a biomarker in esophageal SCC [77].

In summary, the mechanistic map highlights several drug classes—antineoplastics, immunomodulators, phosphodiesterase and kinase inhibitors, and epigenetic modulators—targeting interconnected key pathways in RDEB. Bendroflumethiazide and ibutilide showed the highest impact on map circuits. Although some drugs may have unspecific activities, their identification underscores the model’s ability to capture clinically relevant drugs within this complex condition. In fact, single-agent therapies do not seem to suffice, and combination approaches, including synergistic use with gene or cell therapies, may be required for optimal therapeutic outcomes [2].

### 3.3. Limitations and Translational Considerations

The main limitations of this study include the small cohort size, a common constraint in rare diseases, and the fact that all patients were pediatric and carried PTC variants with severe phenotypes, which precludes its generalizability to adults or milder cases. Despite this homogeneity in the cohort genotype, differences in disease stage, inflammatory status, sex, and age may alter mechanistic circuits. Indeed, restrictive statistical thresholds together with a methodology that provides high-confidence predictions was used. Nonetheless, these findings merit further validation in experimental and clinical settings, focused on testing efficacy in RDEB-relevant circuits and confirming safety. Although the identified drugs target relevant KDT-circuits, some are not highly specific and may produce off-target effects or adverse events. This likely reflects the inherent complexity of RDEB, where multiple intertwined pathways drive disease hallmarks. In addition, the use of whole skin biopsies may dilute some transcriptomic signals. However, these choices preserve a more physiological tissue context compared to isolated cells. Although the use of transcriptomic data is a valid and widely used approach, it does not provide temporal dynamics and therefore molecular responses to wounds and infections are not fully captured. Finally, optimal therapy for RDEB likely requires combination approaches as single-agent treatments seem insufficient. Despite these limitations, the mechanistic map provides meaningful insights into early disease processes and a rational framework for drug repurposing. This is particularly relevant given the rarity and vulnerability of pediatric RDEB patients for whom molecular and clinical data are more limited than in adults.

## 4. Materials and Methods

The experimental design of this study is illustrated in Figure 6.

### 4.1. Study Participants and Ethics Approval

This study was performed using transcriptomic baseline data obtained from skin biopsies of RDEB patients recruited in the MesenSistem-EB clinical trial (NCT04153630 with Registration Date 6 November 2019 and EudraCT 2017-000606-37). Specifically, 8 patients clinically and molecularly diagnosed with RDEB and 6 unaffected individuals were analyzed. The study was conducted in accordance with the Declaration of Helsinki and subsequent revisions (Helsinki, Finland, October 2024) after approval from the Ethics Committee of the Hospital Universitario La Paz (HULP Codes: PI-3439, PI-1359 and PI-1595). Parents or guardians, and minors aged 12–17 years, signed the informed consent form confirming that the collected data could be published.

### 4.2. Molecular and Clinical Characterization of the Cohort

Basic demographic and clinical characteristics of unaffected individuals and RDEB patients are described in Table S1. Molecular diagnosis of EB was performed by antigenic immunomapping and COL7A1 sequencing following recommended methods [78]. Percent of C7 expression (compared to control) was calculated from corrected total fluorescence (CTCF; mean ± SD) in 5 random fields per sample using ImageJ (version 1.54). Circulating C-reactive protein (CRP) levels were measured by standard methods.

Disease severity was determined by the Epidermolysis Bullosa Disease Activity and Scarring Index (EBDASI), which quantifies involvement of skin, scalp, mucous membranes, nails, and other epithelialized surfaces in terms of activity (0–276 points) and damage (0–230 points) [79]. Pruritus was assessed using the Leuven itch scale v1.0 that measures its frequency, intensity, area, and severity. Responses are converted to a 0–100 score, with 100 indicating the worst situation [80].

### 4.3. Transcriptomic Analysis

RNA was obtained from 6 mm diameter skin biopsies from non-affected areas in RDEB patients and unaffected individuals. RNA was extracted using the Qiagen RNeasy Fibrous Tissue kit (Hilden, Germany). RNA concentration and quality were determined with a NanoDrop Spectrophotometer (Thermo Scientific Inc., Waltham, MA, USA) and integrity was verified with a Bioanalyzer 2100 (Agilent, Santa Clara, CA, USA). All samples showed an RNA Integrity Number > 8. cDNA libraries were generated with the Illumina TruSeqRNA sample kit (San Diego, CA, USA) and sequenced using single-end methods (Illumina-NextSeq 1x75, 12 million reads per sample). Raw reads were quality-checked with FastQC and mapped to the reference genome (GRCh38) using TopHat. Reads were normalized using the edgeR package (version 4.5) with the Trimmed Mean of M-values (TMM) method, obtaining log2CPM values for each gene. Raw and processed data are available at the National Center for Biotechnology Information’s Gene Expression Omnibus (accession code GSE119501).

### 4.4. Construction of the RDEB Mechanistic Map

The Bioconductor Hipathia package [81,82] was used as an in silico predictive tool for mechanistic pathway analysis and RDEB map construction. This package uses RNA-Seq gene expression data to infer protein activity levels and estimate circuit activities along Kyoto Encyclopedia of Genes and Genomes (KEGG) signal transduction pathways, predicting the flow of genetic information and regulatory interactions among proteins. At the end of each circuit, protein effectors define the functional profile of cellular processes. The algorithm converts gene expression data into signaling circuit activities and identifies differentially activated circuits (DACs) between patients and controls (FDR < 0.05). The resulting list of KEGG pathways, DACs, and associated effector proteins constitute the RDEB mechanistic map, an actionable in silico model enabling simulations of different scenarios. Map visualization was performed with the R package ggplot2 (version 4.0.1). In addition, an enrichment analysis of dysregulated proteins within the DACs was performed using the Gene Ontology (GO) and Uniprot Keywords databases.

### 4.5. Correlations Between Map Protein Effectors and Key Clinical and Biochemical Parameters

Pairwise correlation analysis was performed with GraphPad Prism 10.2.3 software. The distribution of the data was determined by the Shapiro–Wilk normality test. Statistical significance was assessed by Spearman’s test (nonparametric data) or Pearson’s test (parametric data). *p*-values < 0.05 were considered statistically significant.

### 4.6. Rational Drug Repurposing

The drexml Python package (version 1.1.0) [37] was employed to reposition drugs using the RDEB mechanistic map. Drexml uses multi-output random forest and SHapley Additive exPlanations (SHAP) [83] to prioritize known drug targets (KDTs) that regulate disease map circuits. A bootstrapping validation procedure, based on the Nogueira test (s) [84], was used to assess the prediction performance and KDT stability. Specifically, KDT–circuit pairs were filtered with 95% confidence intervals for performance (R^2^ > 0.6) and Nogueira stability (s > 0.4).

To identify potential drug candidates for RDEB, two strategies were employed. First, the top 30 KDTs with the highest absolute SHAP values were selected and matched with their respective marketed drugs retrieved from DrugBank (version 5.1.10). MBRole software (version 3.0) [85] was used for drug overrepresentation analysis over the Anatomical Therapeutic Chemical (ATC) and KEGG drug classification systems. Secondly, the drug selection process was refined by computing a recalculated score for each drug–KDT–circuit to account for unintended effects across map circuits. This score was calculated as the product of the drexml score sign of the selected KDT, the drug’s effect on the KDT (−1 for inhibition, 1 for activation, 0 otherwise), and the sign of the DACs. By averaging the recalculated score for each drug across the map, a score ranging between −1 (reverses the whole map) and 1 (unintended effects) was obtained. Scores near zero indicated uncertain effects on circuit activity.

Finally, drug categorization combined the mean recalculated score with the impact on the RDEB map by the drug (percentage of circuits influenced by any KDT). Drug categories were defined by K-means clustering (scikit-learn v1.5.2) [86]. The Elbow method (Yellowbrick v1.5) determined the optimal number of clusters, using the distortion score (within-cluster sum of squares) as the metric [87]. This approach allowed objective identification of distinct drug categories based on their combined profile of mean recalculated score and disease map impact.

## 5. Conclusions

This work is the first to use mechanistic modeling to retrospectively explore network-based drug repurposing in EB, mapping the functional disease network of RDEB and linking molecular perturbations to hallmark disease features. The map reliably predicts the activity of key effectors such as IKK1, SMAD7, CASP3, RNASEL, and EIF4EBP, correlating with clinical parameters including CRP, EBDASI, and pruritus scores. Drug repurposing analysis identified 74 marketed drugs targeting the top 30 KDTs (highest SHAP values) and prioritized 16 candidates, encompassing both previously used EB therapies and novel agents. While findings must be interpreted in light of cohort size and lack of RDEB-specific validation, the use of approved drugs accelerates potential clinical translation. Overall, this patient-derived mechanistic map provides a rational framework for drug repurposing, linking molecular perturbations to disease hallmarks and supporting combination or multimodal therapeutic strategies to address the complexity of RDEB. Independently, ongoing studies (e.g., NCT03269474) are using computational approaches to prospectively identify drugs that could enhance wound healing and reduce pain across EB subtypes, reflecting the broader relevance of computational drug repurposing in EB.

## Figures and Tables

**Figure 1:**
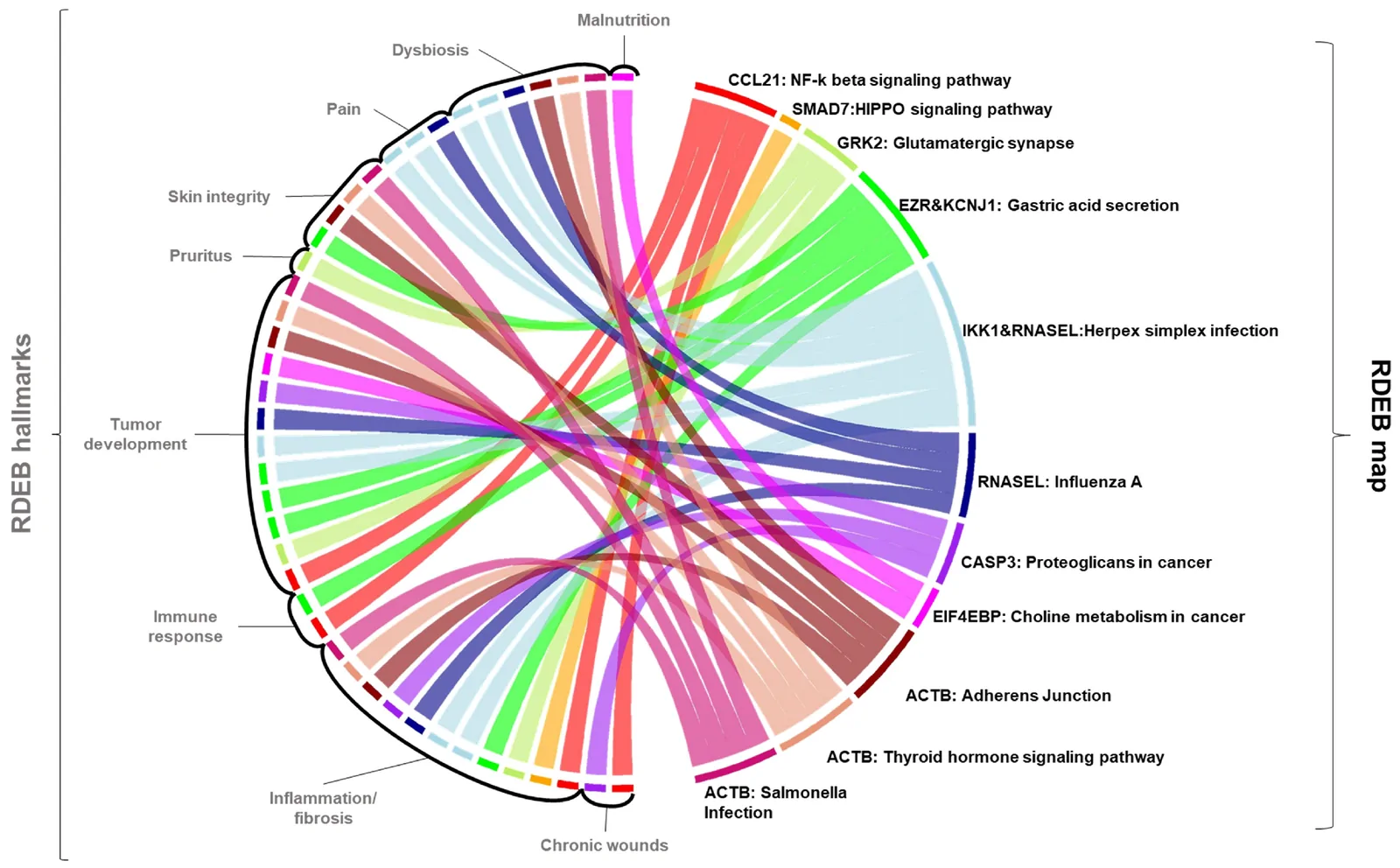
RDEB map effectors linked to disease hallmarks. Effector proteins from the RDEB map are annotated using Kyoto Encyclopedia of Genes and Genomes (KEGG) signaling pathways and linked to well-established RDEB hallmarks. Hallmarks are grouped on the left side of the circle and effectors are shown on the right in different colors connected by lines indicating their functional contributions to each hallmark.

**Figure 2:**
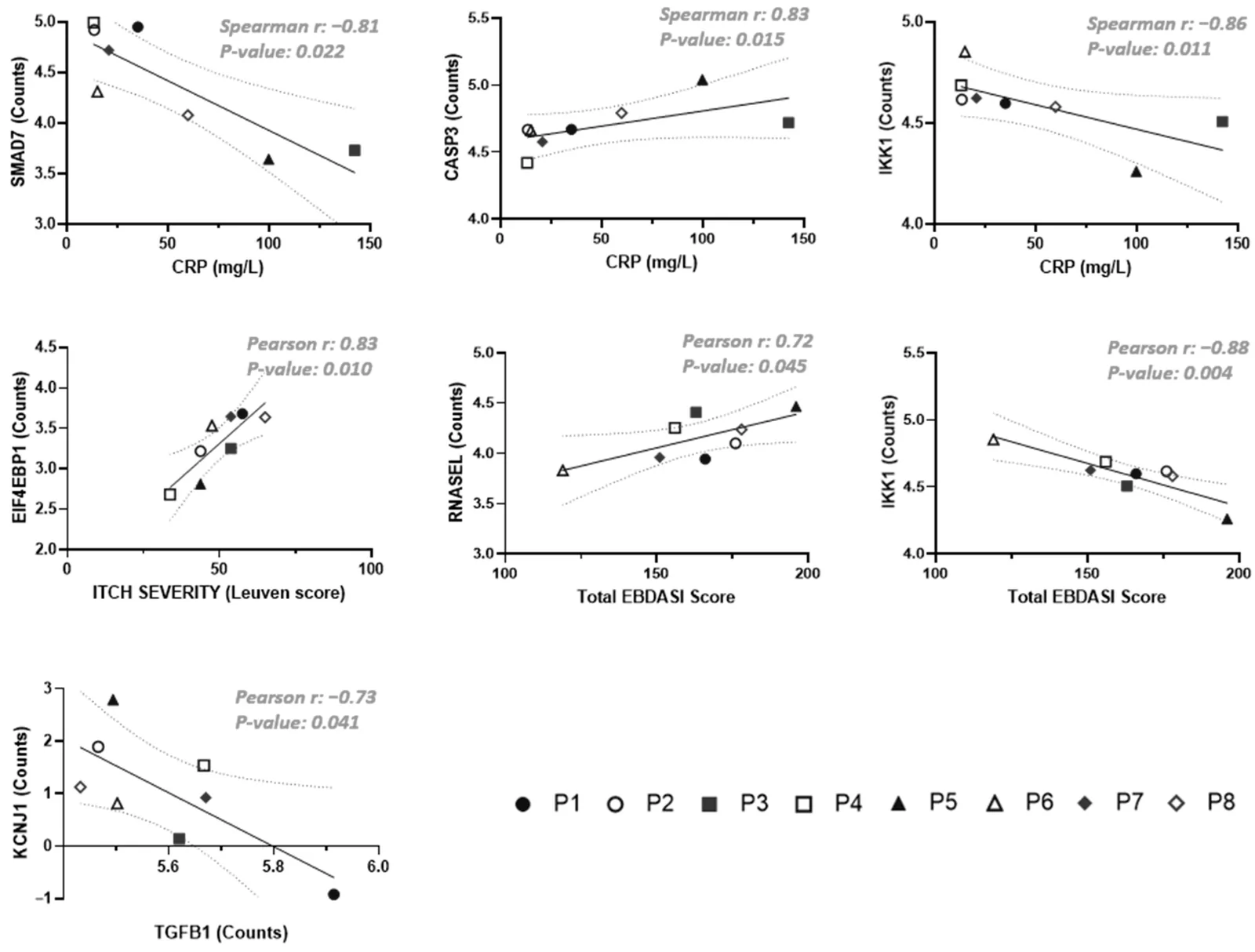
Correlations between patient-derived RDEB map effector expression levels with clinical markers. Statistically significant correlations (*p* < 0.05) between the gene expression (log2CPM counts, normalized by TMM) of RDEB map effectors and clinical measures in the same patients: inflammation (CRP), itch (itch severity scores) and disease severity (EBDASI scores). Correlation coefficients (r) were calculated using Spearman’s or Pearson’s test, as appropriate. Individual data for each patient are represented by different symbols.

**Figure 3:**
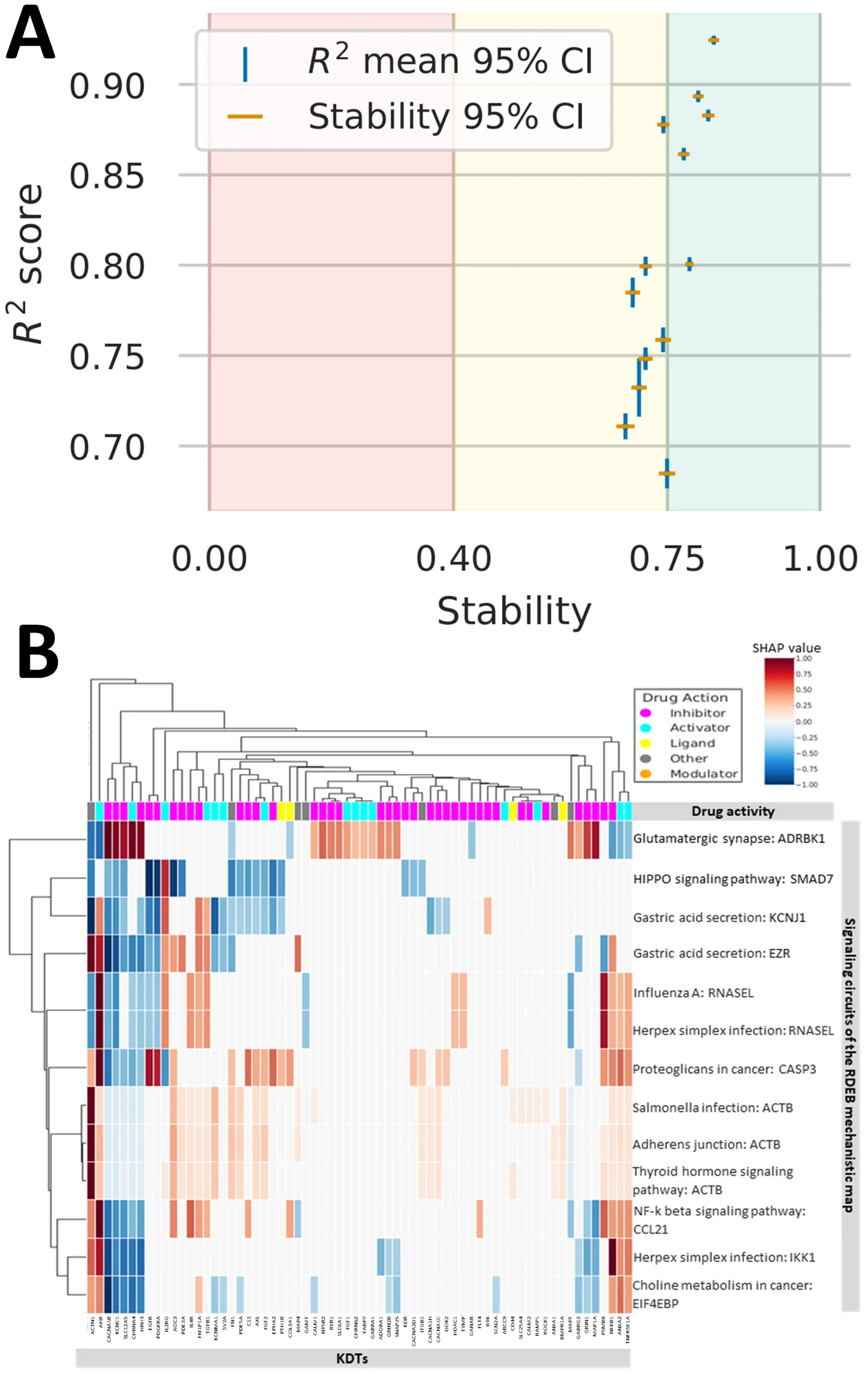
Impact of Known Drug Target (KDT)–circuit pairs on the RDEB map. (**A**) Nogueira stability (s, X-axis) vs. performance (R^2^, Y-axis) for the 13 predicted circuits. All circuits include at least one reliable KDT (CI > 95%, s > 0.4, R^2^ > 0.65). CI = confidence interval. (**B**) Heatmap of 66 KDTs (X-axis) affecting RDEB map circuits (Y-axis). SHAP values (−1 to 1) indicate inhibition (negative) or activation (positive) of each circuit by a KDT, with absolute values reflecting the strength of the effect. Top drug effects for each KDT are displayed above the heatmap.

**Figure 4:**
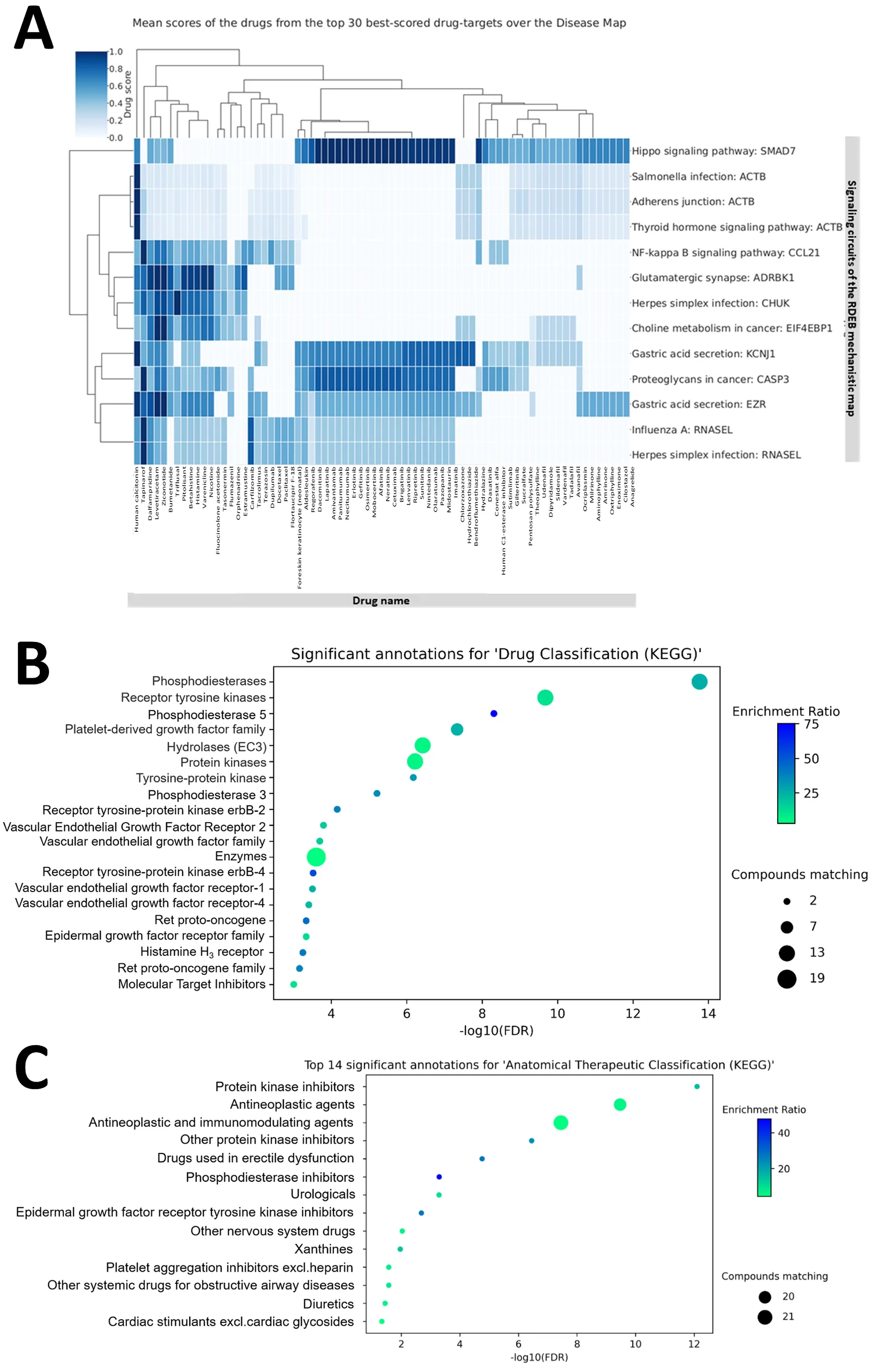
Pharmacological repositioning in RDEB. (**A**) Heatmap of the 74 drugs from DrugBank (v5.1.10) targeting the 30 KDTs with the highest absolute SHAP values. Drugs (X-axis), KDTs in RDEB map circuits (Y-axis), and magnitude of each drug’s impact on the corresponding circuit (blue scale). Most significant (FDR < 0.05) terms are annotated for the 74 approved drugs in (**B**) KEGG and (**C**) ATC classification system. This hierarchical classification system is obtained from https://www.genome.jp/kegg/drug/ (accessed 12 December 2025). Circle size represents the number of drugs, and color (green to blue) indicates the enrichment ratio.

**Figure 5:**
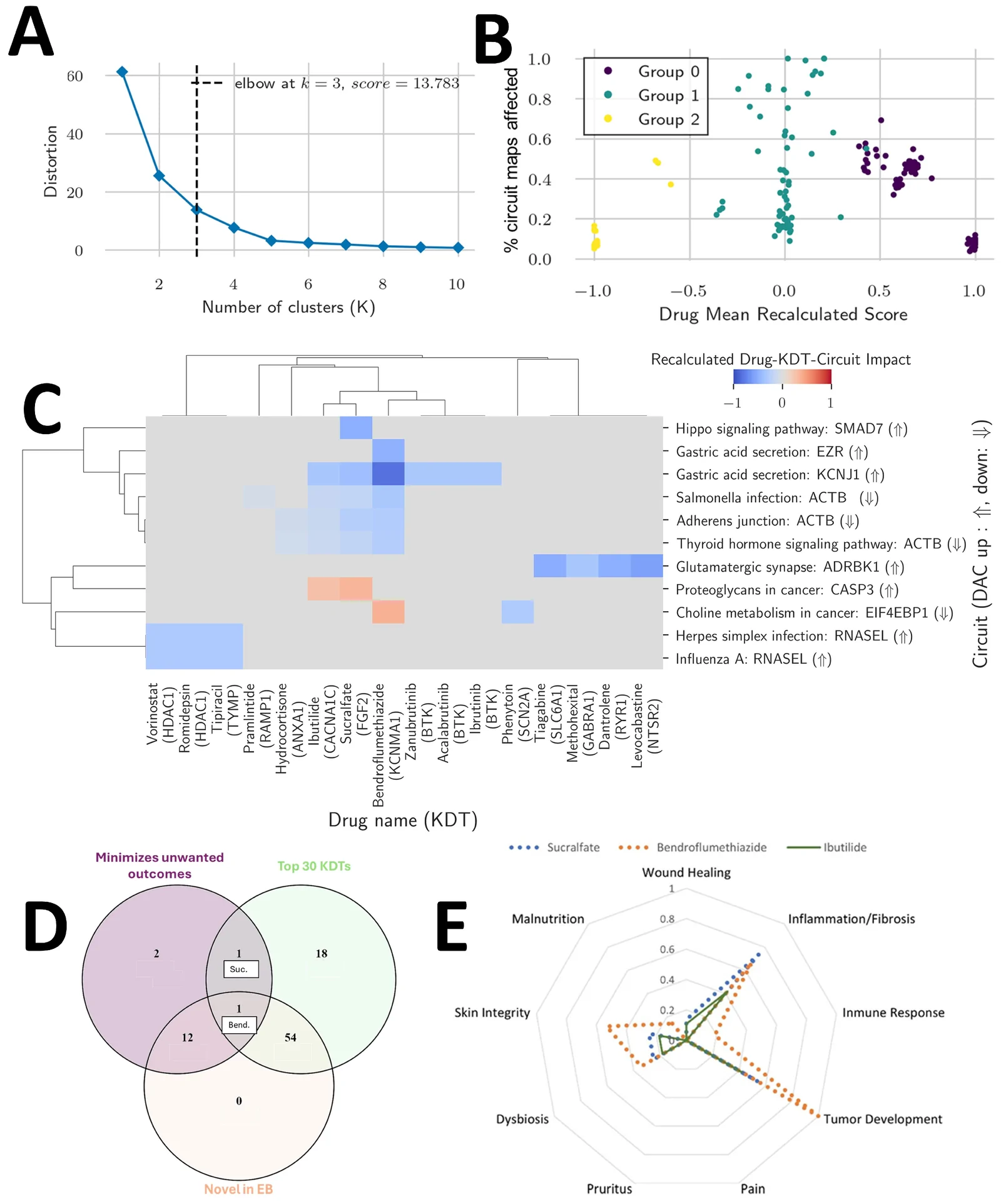
Drug analysis to minimize unintended effects on RDEB map circuits. (**A**) Elbow plot depicting the distortion score for cluster numbers (K) in K-means analysis. The optimal number of clusters is K = 3. (**B**) Scatter plot of individual drugs (points) based on Drug Mean Recalculated Score (lower = better efficacy) and percentage of disease map affected (higher = greater impact). Colors indicate cluster assignment (Groups 1–3). (**C**) Heatmap of drug–KDT–circuit trio. X-axis: drugs selected by recalculated score with their KDT. Y-axis: pathways associated with each pair. (**D**) Venn diagram showing the number of repurposable drugs targeting the top 30 KDTs; those without unintended negative impact the RDEB map circuits and those novel in EB. (**E**) Predicted impact of sucralfate, ibutilide, and bendroflumethazide on RDEB hallmarks, measured as normalized sum of impact strength on the KDT–circuit pairs. Drug information was obtained from DrugBank v5.1.10. Suc.: Sucralfate; Bend.: Bendroflumethiazide.

**Figure 6:**
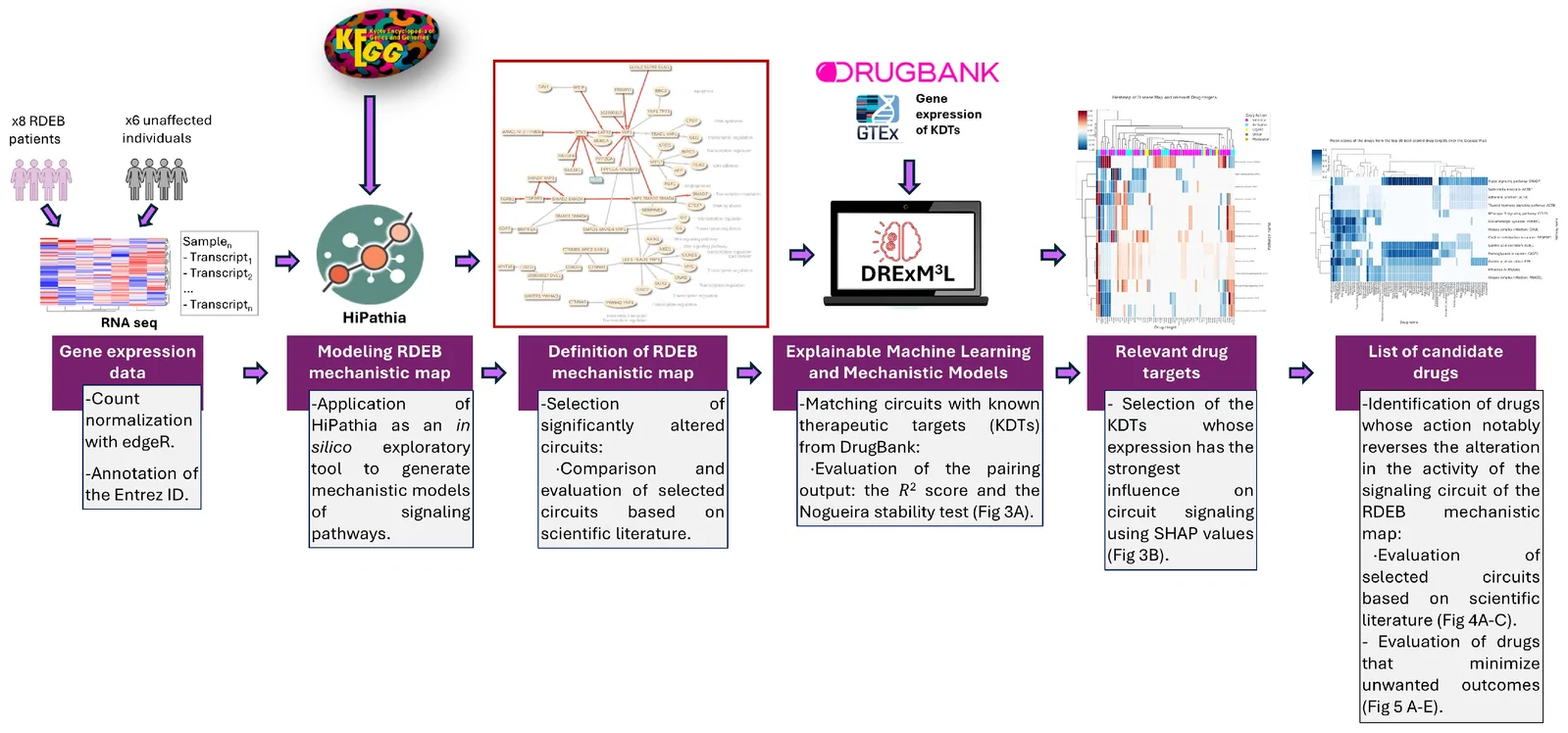
Machine learning-based drug repositioning methodology and map generation in RDEB. The methodology involves the analysis of gene expressions in RDEB skin from RNA-Seq data (available in GSE119501) with Hipathia predicting the activity of altered signaling circuits in the disease. Once built, the RDEB map was used as input data for the drexml-guided determination of repurposed drugs for RDEB.

**Table 1:** Recessive Dystrophic Epidermolysis Bullosa (RDEB) skin mechanistic map.

**ACTIVATED CIRCUITS (RDEB vs.** **HEALTHY)**		
**RDEB KEGG PATHWAY**	**EFFECTOR PROTEIN**	**EFFECTOR ACTIVITY**
NF-kappa β signaling pathway	C-C Motif Chemokine Ligand 21 (CCL21)	Inflammatory Response
HIPPO signaling pathway	Mothers Against Decapentaplegic Homolog 7 (SMAD7)	Transcription Regulation
Glutamatergic synapse	G Protein-Coupled Receptor Kinase 2 (GRk2/BARK1/ADRBK1)	Apoptosis
Gastric acid secretion	Ezrin (EZR)	Cell Shape
	Potassium Inwardly Rectifying Channel Subfamily J Member 1 (KCNJ1)	Potassium Transport
Herpes simplex infection	Nuclear Factor NF-Kappa-β Inhibitor Kinase-α (CHUK/IKK1/IKKα)	Transcription Regulation
	2-5A-Dependent Ribonuclease (RNASEL)	Antiviral Defense
Influenza A	2-5A-Dependent Ribonuclease (RNASEL)	Antiviral Defense
Proteoglycans in cancer	Caspase 3, Apoptosis-Related Cysteine Protease (CASP3)	Apoptosis
**REPRESSED CIRCUITS (RDEB vs.** **HEALTHY)**		
**RDEB KEGG PATHWAY**	**EFFECTOR PROTEIN**	**EFFECTOR ACTIVITY**
Choline metabolism in cancer	Eukaryotic Translation Initiation Factor 4E Binding Protein 1 (EIF4EBP)	Translation Regulation
Adherens Junction	Actin Beta (ACTB)	Cell Adhesion
Thyroid hormone signaling pathway	Actin Beta (ACTB)	Cell Adhesion
Salmonella infection	Actin Beta (ACTB)	Cell Adhesion

Functional profile of altered circuits in KEGG pathways, associated effectors, and their final activity in RDEB skin compared to unaffected controls (FDR ≤ 0.04).

## Data Availability

The datasets generated and analyzed during the current study are available in the Gene Expression Omnibus (GEO) repository: https://www.ncbi.nlm.nih.gov/geo/query/acc.cgi?acc=GSE119501 (accessed on 15 December 2025).

## Supplementary Materials

The following supporting information can be downloaded at https://www.mdpi.com/article/10.3390/ijms27198436/s1.

## Abbreviations

The following abbreviations are used in this manuscript:

AA	Arachidonic Acid
ATC	Anatomical Therapeutic Chemical classification system
CRP	C-Reactive Protein
EB	Epidermolysis Bullosa
EBDASI	The Epidermolysis Bullosa Disease Activity and Scarring Index
FDR	False Discovery Rate
KDT	Known Drug Target
PDGFR	Platelet-Derived Growth Factor
RDEB	Recessive Dystrophic Epidermolysis Bullosa
RNA-Seq	RNA Sequencing
SCC	Squamous Cell Carcinoma
SHAP	SHapley Additive exPlanations
TMM	Trimmed Mean of M-values
VEGFR	Vascular Endothelial Growth Factor Receptor
